# The benefits of guided imagery on athletic performance: a mixed-methods approach

**DOI:** 10.3389/fpsyg.2025.1500194

**Published:** 2025-04-11

**Authors:** Katrina Volgemute, Zermena Vazne, Romualdas Malinauskas

**Affiliations:** ^1^Latvian Academy of Sport Education, Rigas Stradins University, Riga, Latvia; ^2^Department of Physical and Social Education, Lithuanian Sports University, Kaunas, Lithuania

**Keywords:** athletic performance, cluster analysis, imagery profile, psychological intervention, psychological preparation

## Abstract

**Introduction:**

Imagery as a psychological skill in sports occupies an essential place in the psychological preparation of athletes and is one of the key factors in realizing an athlete’s potential in competitive sports. It’s role in athlete performance, as well as the differences in its use across various sports and demographic groups, has been a compelling topic in both sports’ science and psychology. This topic has remained relevant in recent years.

**Aim:**

This quantitative survey-based and experimental design study aims to first, to determine whether differences in imagery ability profiles, identified through cluster analysis, can characterize differences among athletes with varying levels of athletic achievement. A secondary aim of the study is to evaluate the effect of a guided imagery intervention on imagery abilities and athletic performance.

**Methods:**

A total of 500 athletes from different sports, aged *M* = 21.01 (SD = 2.82), both male and female, completed the Sport Imagery Ability Questionnaire (SIAQ) and provided information regarding their demographics and athletic achievements. In the experimental part of the study, nine alpine skiers were selected to participate in a six-month guided imagery intervention alongside their regular trainings on the ski track to assess the intervention’s impact on performance.

**Results:**

The results of K-means cluster analysis on athletic achievements of athletes showed a four-cluster solution that hat emphasized distinctions between the groups and reduced variation within each group. A Multiple Analysis of Covariance revealed that the four cluster groups differ in their imagery abilities. Pre- and post-intervention assessments for the nine alpine skiers were conducted using SIAQ and control training sessions, revealing significant increase in both imagery ability scores and performance indicators (*p* < 0.05).

**Conclusion:**

The research results support recent findings that athletes with higher athletic achievement tend to have stronger imagery abilities. The significant differences observed between the clusters based on athletic achievement levels were confirmed. By using imagery profiling of athletes with an analytical method, this study provides valuable insights into the role of imagery in athletic success, suggesting that tailored imagery training could enhance performance across different levels of athletic achievement.

## 1 Introduction

Imagery plays a vital and well-recognized role in the training process and sports achievements of athletes, earning its place in both sports practice and sports science. It is acknowledged as one of the most crucial psychological skills, aiding athletes in psychological preparation for competition and enabling them to realize their full potential in critical and stressful situations. It has long been understood that an athlete’s success and performance are significantly influenced by their psychological state and their ability to effectively manage various cognitive processes (Siekańska et al., 2021). Imagery is also one of the most widely utilized psychological tools, proven effective in enhancing sports skills, improving technical performance, psychological preparation, and even facilitating rehabilitation after sports injuries (Gupta et al., 2023; Hartani and Yang, 2023; Gulanes et al., 2024). Previous studies have highlighted the effectiveness of imagery in the training process by emphasizing its significant contribution to enhancing athletic performance (Lin et al., 2021; Lindsay et al., 2023). Through the use of an imagery, athletes can effectively manage the emotions and stress associated with competitive sports environments (Lee et al., 2023). Additionally, by rehearsing various scenarios and strategies in imagery, athletes can better prepare for the physical, technical and tactical demands of upcoming performances. This approach allows for more optimal preparation and can lead to improved outcomes in competition. Based on empirical evidence and scientific literature, this study aims to shed light on the complex relationship between athletes’ imagery abilities and their sports achievements. By identifying the imagery profile characteristics of athletes who have reached different competitive levels, the study presents opportunities for future research and contributes to a deeper understanding of how individualized practical psychological skills interventions can be developed and implemented in practice of sport coaches, specialists and psychologists.

### 1.1 Literature review

The psychological skills are crucial to their overall performance and results. Among these, the power of imagery stands out as one of the most significant contributors. Imagery enhances athletes’ mental resilience and boosts their self-confidence (Welis et al., 2024; Hartani and Yang, 2023). It involves the mental process of vividly reliving a past experience or creating a new scenario to prepare as effectively as possible for an upcoming event (Munroe-Chandler et al., 2023). By simulating reality in such detail, athletes can generate a strong sense of authenticity in their imagined scenarios, leading to more effective outcomes in real-life performance.

Numerous approaches and theories have been developed to explain how imagery works, with most practical approaches emphasizing the importance of engaging all human senses to create a vivid and lifelike mental experience. The various models of imagery underscore the crucial role that imagery and its associated abilities play in enhancing and promoting athletic performance. For instance, the applied model of deliberate imagery use is one of the most recent and widely recognized theoretical frameworks in this area. This model, which represents a significant advancement in imagery research since the original conceptual model was introduced, centers on the concept of conscious imagery, activated when an athlete deliberately envisions a specific goal. It incorporates the components of ‘where,’ ‘when,’ and ‘why,’ which are essential for understanding how imagery is used in sports contexts. Additionally, this theoretical model serves as the foundation for the development of the Sport Imagery Ability Questionnaire (Williams and Cumming, 2011), a highly regarded research instrument that assesses an athlete’s imagery abilities and is widely utilized in sports psychology research.

Within the scientific literature on imagery in sports, two key variables are often emphasized: the ability to imagine and the use of imagery (Budnik-Przybylska et al., 2023). Empirical research suggests a positive correlation between these two factors, indicating that athletes who have a strong ability to imagine are also more likely to effectively use imagery in their training and competition (Dello Iacono et al., 2021). The use of imagery has been positively linked to athletic performance, with studies demonstrating that athletes who excel in using their imagery tend to perform better than those with lower imagery abilities (Lee et al., 2023).

Psychological skills training, including imagery, plays a key role in fostering athletes’ overall performance. Imagery can be used for a variety of purposes in the training process, such as improving physical techniques, correct mistakes, and promoting various psychological aspects, such as focus and confidence (Guillot et al., 2024; Lin et al., 2021; Bedir and Erhan, 2021). Research on the effectiveness of imagery in sports has consistently shown that imagery and its associated functions or abilities have a significant and mutually beneficial relationship with athletes’ performance across various aspects. For example, a study by Hartani and Yang (2023) found that imagery focused on developing athletic abilities and technical skills significantly enhances skill acquisition and performance, which is a critical component of the athlete training process. However, recent research has highlighted also the complex relationship between imagery skills and athletic performance (Ladda et al., 2021). While some studies suggest that athletes with higher imagery skills tend to benefit more from imagery training, leading to enhanced performance outcomes, others indicate that imagery skills alone are not a consistent predictor of performance improvements (Lindsay et al., 2023; Bedir and Erhan, 2021). These inconsistencies highlight the need for further investigation into the factors that facilitate imagery interventions in sports.

Another crucial aspect of imagery in sports is its role in enhancing motivation. To effectively boost an athlete’s motivation, it is important to set clear goals that serve this motivating function. Imagery contributes to motivation by enabling athletes to visualize their goals and outcomes successfully. Research indicates that goal imagery has a significant positive relationship with performance. The ability to vividly imagine an ideal performance and its successful outcome increases confidence during competition, ultimately leading to improved performance (Aikawa and Takai, 2021).

Moreover, the ability of imagery to incorporate emotions equips athletes with essential self-regulation skills and serves as a foundation for maintaining physical self-efficacy. This ability supports athletes in understanding and managing the emotions they experience during sports. Such emotional imagery helps them mentally prepare for future competitions, enhancing their readiness to realize their potential and achieve high levels of success (Watt and Morris, 2020; Kouali et al., 2020). By employing mastery imagery ability, athletes can visualize themselves successfully executing skills in a competitive scenario. The mastery imagery ability serves the general motivational function of imagery. Its purpose is to develop the aspect of imagery ability, which promotes athletes’ self-confidence and helps athletes overcome challenges in the sports environment by maintaining their confidence in their abilities or self-efficacy (Cherappurath et al., 2020; Williams et al., 2021; Morone et al., 2022).

The success of imagery interventions largely depends on how effectively they are tailored to the individual athlete. Given that athletes’ imagery capabilities can vary significantly, it is crucial to adopt a personalized approach that addresses their specific needs. Therefore, further research is needed to explore the specific aspects of imagery that are most closely linked to clear and measurable athletic achievements.

### 1.2 Study aims and hypothesis

This study has two primary aims: first, to explore the relationship between imagery ability and athletic achievement among athletes at various levels. Through cluster analysis, this study aims to identify and characterize distinct imagery profiles among athletes with varying levels of success. Secondly, to evaluate the impact of individualized guided imagery intervention on athletes’ different imagery ability, as well as on their athletic performance in alpine skiing. By incorporating guided imagery sessions into their training process, this study aims to enhance both the athletes’ imagery abilities and their athletic performance.

**Figure 1:**
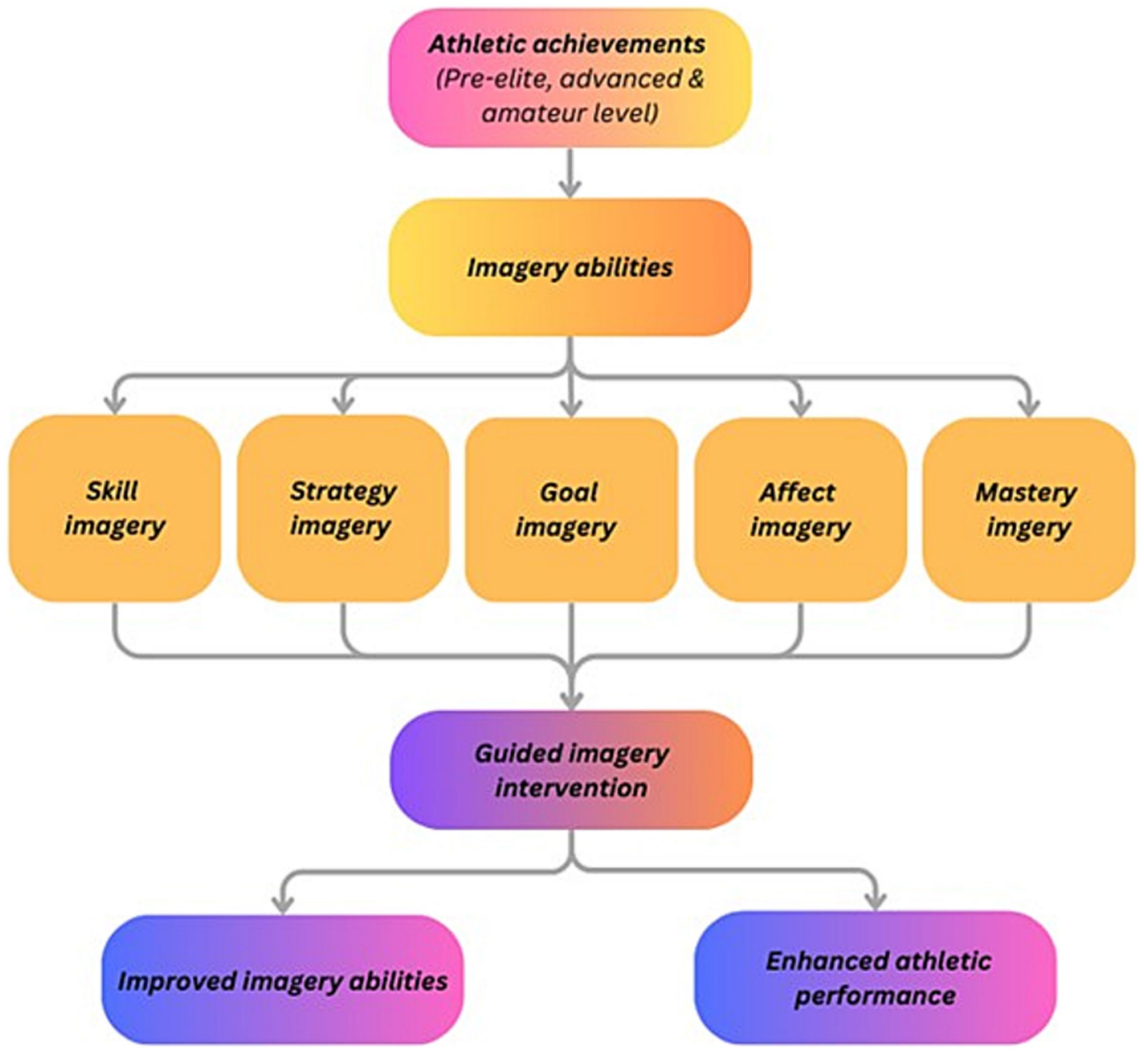
Research framework and hypotheses.

Based on existing literature and previous research on imagery in sports, it is hypothesized that athletes’ levels of athletic achievement have a significant and positive relationship with their imagery abilities. Furthermore, it is suggested that integrating a six-month guided imagery intervention into the training process will enhance both athletic performance and imagery abilities. Specifically, strategy imagery (focused on tactical planning and decision-making) and affect imagery (focused on emotional regulation) are expected to show statistically significant improvements in relation to athletic performance. One key theoretical model supporting this is the PETTLEP model, developed by Holmes and Collins (2001), which emphasizes that incorporating guided imagery into realistic practice can effectively enhance athletic performance. Similarly, Cumming and Williams (2012) proposed an applied model of deliberate imagery use in sport, reinforcing the role of imagery in improving athletic performance. Other recent studies have highlighted the specific connections between different types of imagery abilities and improvements in athletic performance or skill development (see Figure 1).

The use of imagery to enhance athletic performance is highly relevant topic in today’s sport science (Janjigian, 2024; Williams et al., 2021). Only few studies have explored the relationship between individual athletes’ imagery abilities and their performance (Yadolahzadeh, 2020; Lu et al., 2020). Investigating athletes’ imagery profiles using advanced analytical methods like cluster analysis can offer a comprehensive approach to developing tailored imagery interventions based on individual differences. This approach can reveal how athletes employ various imagery abilities at different levels of sport and provide valuable insights into how to effectively incorporate imagery into both training and competition environments.

## 2 Materials and methods

### 2.1 Participants

Before selecting the study sample, its size was determined using G*Power 3.1.9.6 software, with a 5% *α*-error level and an 80% power level (which corresponds to a β-error rate of 20%). The analysis indicated that at least 147 athletes were required for each athlete level group. Participants for the first part of the research were selected using random sampling methods. The research sample consisted of 500 athletes, aged *M* = 21.01 years (SD = 2.82), comprising both female (*n* = 216) and male (*n* = 284). The athletes represented a total of 62 different individual and team sports. The distribution per sport was as follows: basketball (*n* = 75), football (*n* = 57), track and field (*n* = 41), volleyball (*n* = 36), hockey (*n* = 33), fitness (*n* = 20), artistic gymnastics (*n* = 20), floorball (*n* = 19), swimming (*n* = 18), karate (*n* = 12), rowing (*n* = 10), boxing (*n* = 10), alpine skiing (*n* = 9), sports dancing (*n* = 8), gymnastics (*n* = 8), orienteering (*n* = 7), judo (*n* = 6), frisbee (*n* = 6), handball (*n* = 6), weightlifting (*n* = 6), taekwondo (*n* = 6), and others (*n* = 90).

The average experience of the athletes in competitive sports was *M* = 10.01 years (SD = 4.27). All athletes included in the national team had competition experience at the national or international level. Their levels of achievement varied, ranging from winning places in World Championships, European Championships, or National Championships to participation in national level competitions.

In the second part of this research nine youth alpine skiers age *M* = 18.22 years (SD = 1.17) participated in a guided imagery intervention aimed at assessing the impact of imagery ability on athletic performance. This part of the research employed convenience sampling to recruit athletes based on specific inclusion criteria. The inclusion criteria for participation were as follows: athletes engaged in regular alpine skiing training, with at least four to five sessions per week during the competitive season. These training sessions included a combination of on-snow, physical conditioning, and technical skill development, ensuring well-rounded preparation for the athletes. All participants had competed at both national and international levels (such as competitions organized by the International Ski Federation), which provided them with broad exposure to varying levels of competition, making them suitable participants for this study. Each athlete had a consistent training history, without any significant interruptions over an extended period. Additionally, it can be noted that all participants were without any major injuries at the time of the study that could have impacted their performance results. The exclusion criteria were that the participant did not meet the above specified criteria or did not agree to participate in the intervention program. Ultimately, nine athletes agreed to participate in the guided imagery intervention. These athletes were categorized as pre-elite or advanced-level competitors. The final sample consisted of five female and four male athletes. The participants had considerable experience in alpine skiing, with an average of 8.77 years (SD = 2.25) of active involvement. All athletes were actively competing at both the Latvian and international levels, with a weekly training load of 5–6 sessions.

### 2.2 Measures

Athletes’ imagery abilities were assessed using the Latvian-adapted version of the Sport Imagery Ability Questionnaire (SIAQ; originally developed by Williams and Cumming, 2011; adapted into Latvian by Volgemute et al., 2019). The previously adapted Latvian version demonstrated good internal consistency and reliability, with Cronbach’s alpha values ranging from 0.66 to 0.87 across all imagery ability scales (Volgemute et al., 2019). This measurement tool is recognized as a valid and reliable method for assessing various aspects of athletes’ imagery characteristics.

The SIAQ is a self-assessment questionnaire comprising 15 items, divided into five subscales that evaluate different types of imagery abilities: (1) skill, (2) strategy, (3) goal, (4) affect, and (5) mastery. The SIAQ items are evaluated using a 7-point Likert scale. Sample items include “I work with 100% effort even when mistakes are made in the performance of the competition, and everything does not go smoothly” (strategy imagery ability) and “Experienced feelings and emotions that arose from the results of the competition activity” (affect imagery ability).

To achieve the secondary aim of the study and gain deeper insight into how imagery abilities affect sports performance, both the athletes’ imagery abilities and the sports performance indicators of nine alpine skiers were evaluated. During the experimental phase, athletes performed three control sessions in addition to the guided imagery intervention. In these control trainings, the Microgate Pro Kit Timing time device was used to determine the time each athlete took to successfully perform the ski slalom course.

### 2.3 Design and data collection procedure

The main part of this quantitative survey-based and experimental design study was conducted over a two-year period, beginning in May 2021 and concluding in April 2023. Data were collected from athletes across various sports and achievement levels (*n* = 500) using paper forms of the Sport Imagery Ability Questionnaire (SIAQ) distributed to participants. The target population included athletes who were actively engaged in their respective disciplines. Athletes’ participation in the study was voluntary. Athletes were informed about the anonymity of their data. The research was conducted after coordination with the guidelines of the Ethics Committee of the Latvian Sports Education Academy (No. 4/51813).

#### 2.3.1 Guides imagery intervention

In the study experiment, the alpine skiing athletes’ (*n* = 9) imagery abilities were assessed using the same SIAQ measurement tool. Based on these initial assessments, a series of guided imagery intervention sessions was designed specifically to enhance the imagery abilities of alpine skiers. This intervention was then integrated into their training process throughout the training season, with the content structured around the key scales of the SIAQ: skill, strategy, goal, affect, and mastery imagery abilities. Guided imagery sessions were conducted over a six-month period. This time period was chosen to cover one full season of active training and competition for alpine skiers. It also ensures that the effects of the guided imagery intervention on imagery abilities and the control of training sessions can be accurately assessed. Each session lasted not less than 20 min. The time limit for guided imagery sessions was not restricted. The duration was highly dependent on the athlete’s own feelings, as well as their ability to maintain focus during the guided imagery exercises, ensuring that each session could achieve the most effective results. Cumming and Williams (2012) emphasize that the effectiveness of imagery training is influenced by its frequency, duration and consistency. Their research suggests that regular but manageable imagery sessions, such as two sessions per week, can effectively enhance athletic performance by reinforcing neuromuscular pathways and cognitive processes without causing cognitive fatigue. Furthermore, imagery training sessions are generally recommended to be kept within 30 to 40 min, making a 20-min duration an appropriate and effective choice (Cumming and Williams, 2012; Amasiatu, 2013).

A total of 47 guided imagery sessions were conducted two times per week. The intervention included a variety of imagery scenarios, such as sensory movement visualization, where athletes imagined the sensations of skiing to mentally replicate the physical experience. A key component of the training was time control, where athletes compared the actual time taken to complete the ski track with the time spent mentally rehearsing the course, aiming to align mental and physical performance. Additionally, gate layout visualization exercises were practiced, in which athletes visualized the placement of the gates on the training track either before or after training, reinforcing their spatial awareness and course memorization. The intervention also included guided imagery scenarios where athletes visualized themselves performing specific exercises during training, standing at the starting line while experiencing the emotions associated with competition, or imagining a flawless performance. Furthermore, competition simulations were conducted through imagery, emphasizing the use of all senses to create a vivid and realistic mental experience. These exercises were designed to enhance the athletes’ psychological preparedness and overall performance by integrating sensory-rich imagery into their regular training routine. In addition to the guided imagery exercises, athletes were introduced to the theoretical and practical aspects of imagery to further enhance its application in their sport.

To assess the development of imagery abilities and their impact on athletic performance, the study included three control training sessions: one before the implementation of the guided imagery intervention, one midway through the study, and one final session at the end. These control sessions aimed to evaluate the effectiveness of the imagery intervention on the athletes’ performance. During each session, the athletes’ times were recorded for their first and second successful runs on a slalom course. In each of the control trainings, the athlete’s task was to perform the designated slalom course on the slope in the shortest possible time. It is important to mention that all sessions took place in a closed ski arena, ensuring consistent conditions for each control session. Such conditions were very important, as even the smallest changes can have a significant impact on athletes’ performance on slalom ski track, where results are often determined by hundredths of a second. At the end of the study, the athletes’ imagery abilities were evaluated again by using SIAQ. Results were then compared with data collected at the beginning of the study to assess the effectiveness of the guided imagery intervention.

### 2.4 Statistical analysis

The statistical analysis of the data obtained in this study was performed using IBM SPSS Statistics software, version 28.0, for the Windows operating system. In the initial step, 546 questionnaires were collected; however, 46 were deemed invalid, primarily due to incomplete responses. For the subsequent analysis, 500 valid questionnaires were retained (Volgemute, 2025). Statistical methods were selected according to the main objectives of the study. Descriptive statistics were analyzed for all questionnaire data, including demographic information and SIAQ responses.

Based on the aims of the study, the appropriate mathematical statistical analyzes were selected. To assess data normality, skewness and kurtosis coefficients were evaluated. Values of skewness and kurtosis between −1 and 1 were considered to indicate a normal distribution of the data. Cronbach’s alpha was used to assess the internal consistency of the SIAQ scales. A coefficient value of 0.6 or greater was considered acceptable. In order to investigate the correlations between athletes’ imagery ability (skill, strategy, goal, affect, mastery imagery abilities), sport (individual or team sports), age, gender and athletic achievements (pre-elite level, senior level or amateur level athletes), bivariate Pearson correlation analysis was used. Multivariate Analysis of Covariance (MANCOVA) was utilized to test the hypothesis that athletes with higher levels of athletic achievement would have higher scores in their imagery abilities. In MANCOVA, athletic achievement levels served as the fixed factor, while SIAQ imagery abilities scores were the dependent variables. Gender, age, and sport type were included as covariates.

In the final step of the results analysis, K-means cluster analysis was employed to classify athletes based on their athletic achievement scores from the SIAQ. This clustering method allowed for the creation of distinct imagery ability profile groups, enabling a more detailed examination of the relationships between athletes’ imagery abilities and their levels of athletic achievement. The Euclidean distance metric was used for clustering, and a four-cluster solution was selected based on within-cluster variance and participant distribution to ensure meaningful differentiation.

The same Latvian language version of the SIAQ was used to measure the imagery abilities of the alpine skiers (*n* = 9) who participated in the guided imagery intervention sessions. Descriptive statistics of the athletes’ imagery abilities before and after the experimental phase were compared and analyzed. Since the obtained data did not follow a normal distribution, non-parametric methods were chosen to reliably analyze changes in both imagery and control training session indicators. The Wilcoxon signed rank test was selected to assess the reliability of average changes before and after the guided imagery intervention. The results were interpreted based on the *p*-value, with a significance level set at *p* ≤ 0.05.

## 3 Results

### 3.1 Descriptive statistics

Table 1 summarizes the means, standard deviations, skewness, kurtosis, and data reliability of SIAQ data collected from athletes. The mean scores indicate that across the sample, the lowest scores are for mastery imagery ability (*M* = 4.97, SD = 1.02). These imagery abilities are characterized by athletes’ ability to create images of being in control of situations or coping with difficult scenarios. Athletes often use these imagery abilities to control emotions and regulate anxiety. Similarly, low scores are observed for strategy imagery ability (*M* = 5.07, SD = 0.98), which are related to athletes’ use of imagery in competitive situations and their ability to create mental images of different strategies during competitions.

**Table 1:** Descriptive statistics, normality test, and internal consistency results of SIAQ scales (*n* = 500).

Factor	Descriptive scores				Cronbach’s alpha (α)
	*M*	*SD*	*Sk*	*Ku*	
SIAQ					
F1: Skill imagery	5.66	0.89	−0.49	0.01	0.855
F2: Strategy imagery	5.07	0.98	−0.37	−0.24	0.843
F3: Goal imagery	5.36	1.15	−0.58	−0.18	0.846
F4: Affect imagery	5.78	0.85	−0.81	1.36	0.862
F5: Mastery imagery	4.97	1.02	−0.28	−0.26	0.844
F6: Global imagery	5.37	0.73	−0.21	−0.58	0.797

SIAQ, Sport Imagery Ability Questionnaire; M, Arithmetic Mean; SD, Standard Deviation; Sk, Skewness; Ku, Kurtosis.

In contrast, the highest scores across the athletes’ sample are in affect imagery ability (*M* = 5.78, SD = 0.85), followed by skill imagery ability (*M* = 5.66, SD = 0.89). The affect imagery ability are strongly connected with the emotions that arise from sports situations. This ability can be described as the capacity to create self-imagery that reflects different feelings and emotions. This is a crucial factor in making imagery vivid and experiencing imagined situations as if they were happening in real life. Skill imagery ability are described by athletes’ ability to visualize scenarios connected with sports skills, such as seeing the performance of a technical element in training. Goal imagery scores are moderate (*M* = 5.36, SD = 1.15), reflecting the athletes’ ability to easily visualize their achievements. Global imagery ability (*M* = 5.37, SD = 0.73) are calculated by summarizing all the scales together and represent the overall skill of athletes in visualizing different scenarios.

The obtained data confirm a normal distribution, as indicated by the skewness values (ranging from −0.81 to −0.21) and kurtosis values (ranging from −0.58 to 1.36). Nearly all parameters fall within the range of −1 to 1 for both skewness and kurtosis, with the exception of the kurtosis value on the affect imagery ability scale. As George and Mallery (2010) point out, when the skewness and kurtosis values are between 2 and −2, the distribution of all variables is not significantly different from a normal distribution and all parametric statistical criteria can be applied.

The reliability analysis of the results was carried out using Cronbach’s alpha coefficient, which showed high internal consistency indexes across all imagery scales, with coefficients ranging from 0.797 to 0.862. All the scales’ coefficients exceed the accepted value (*α* = 0.70) in social science studies, indicating strong reliability and consistency of the measurements.

### 3.2 Correlation analysis

The Pearson correlation results are displayed in Table 2. The obtained results indicate a significant relationship between the factors under analysis. Pearson’s correlation coefficients were estimated according to guidelines in the social sciences (Cohen, 1988). Correlation is considered weak if *r* > 0.1, medium if *r* > 0.3 and strong if *r* > 0.5. Further evaluating the athletes’ achievements, the research sample (*n* = 500) was divided into three groups according to individual highest achievements: pre-elite level athletes, advanced level athletes and amateur level athletes. In the context of this study, athletes who competed in the national, Baltic, European or World championships and won prize-winning places, as well as those who reached the master class level in their sport, were considered pre-elite level athletes. The advanced level athletes were categorized those athletes who have mostly competed in junior national or international competitions or have competed in national or Baltic Championships without winning award-winning places. Amateur level athletes, the largest group, consisting mostly of university athletes who actively compete in various levels of competitions but have not reached the high achievement levels of pre-elite level or advanced level athletes.

**Table 2:** Pearson correlation matrix between sport type, SIAQ scales, age, gender and achievements.

Factor		1	2	3	4	5	6	7	8	9	10
1	Sport type	1									
2	Skill imagery	−0.124*	1								
3	Strategy imagery	−0.033	0.386**	1							
4	Goal imagery	0.032	0.394**	0.516**	1						
5	Affect imagery	−0.046	0.419**	0.314**	0.387**	1					
6	Mastery imagery	0.016	0.389**	0.550**	0.482**	0.345**	1				
7	Global imagery	−0.035	0.686**	0.764**	0.788**	0.644**	0.765**	1			
8	Age	−0.049	−0.023	−0.016	−0.078	−0.019	−0.108*	−0.070	1		
9	Gender	221**	−0.085	−0.007	0.050	−0.141**	0.013	−0.036	0.101*	1	
10	Achievements	0.144**	−0.110*	−0.108*	−0.177**	−0.114*	−0.083	−0.163**	0.003	−0.013	1

**Correlation is significant at the 0.01 level (two-tailed); *Correlation is significant at the 0.05 level (two-tailed).

Based on the obtained results, it can be concluded that there are significant and strong relationships between all the imagery scales. Additionally, sport type has a significant relationship only with skill imagery ability (*r* = −0.124, *p* < 0.05), indicating that team sports athletes have higher skill imagery ability than individual sports athletes. Athlete age showed relationships only with mastery imagery ability (*r* = −0.108, *p* < 0.05), suggesting that younger athletes tend to have stronger mastery imagery ability. Regarding gender, no significant relationship can be concluded with imagery abilities except for affect imagery ability (*r* = −0.141, *p* < 0.01). This indicates that female athletes have stronger imagery abilities related to emotional experiences in sports. No other significant relationships between imagery abilities and either gender or sport type were found.

This research aimed to explore how athletes’ achievement levels relate to their imagery abilities. Pearson correlation results indicate that athletic performance has a statistically significant correlation with almost all scales of SIAQ. The only exception is mastery imagery (*r* = −0.083, *p* > 0.05) ability. Athletic achievements have a significant relationship with skill imagery (*r* = −0.110, *p* < 0.05), strategy imagery (*r* = −0.108, *p* < 0.05), goal imagery (*r* = −0.177, *p* < 0.01), affect imagery (*r* = −0.114, *p* < 0.05) and global imagery (*r* = −0.163, *p* < 0.01) ability. These results suggest that higher achievement level athletes tend to have higher levels of imagery.

### 3.3 Multiple analysis of covariance (MANCOVA)

MANCOVA was conducted to examine the differences between athletes’ achievement levels and their imagery abilities. Table 3 presents the results of these differences from the athlete sample. The Pillai’s Trace of the MANCOVA was 0.045 (*F* (2, 497) = 1.876, *p* < 0.05), indicating that the overall main effect of athletes’ achievements reached statistical significance. Athletes with higher levels of athletic achievement generally have higher imagery ability across various types of imagery. Among the 500 athletes in the research sample, 34.2% (*n* = 171) were categorized as pre-elite level athletes, 23.2% (*n* = 116) as advanced level athletes, and 42.6% (*n* = 213) as amateur level athletes. The analysis revealed significant differences between athletic achievement levels and several types of imagery abilities: skill imagery (*F* (2, 497) = 3.029, *p* < 0.05, *η*_p_^2^ = 0.012), strategy imagery (*F* (2, 497) = 3.914, *p* < 0.05, *η*_p_^2^ = 0.015), goal imagery (*F* (2, 497) = 8.087, *p* < 0.05, *η*_p_^2^ = 0.032), affect imagery (*F* (2, 497) = 3.367, *p* < 0.05, *η*_p_^2^ = 0.013), and global imagery (*F* (2, 497) = 6.916, *p* < 0.05, *η*_p_^2^ = 0.026). The only exception was mastery imagery ability, which did not show significant differences between the three groups of athletes. This suggests that mastery imagery might not be as strongly associated with athletic achievement levels.

**Table 3:** Multivariate Analysis of Covariance of SIAQ scales and athletes’ achievements level.

Factor	Pre-elite level (*n* = 171)	Advanced level (*n* = 116)	Amateur level (*n* = 213)	*F*	*η* ^2^
F1: Skill imagery	5.78 (0.86)	5.68 (0.83)	5.56 (0.89)	3.029*	0.012
F2: Strategy imagery	5.18 (0.88)	5.23 (0.92)	4.93 (0.99)	3.914*	0.016
F3: Goal imagery	5.63 (1.03)	5.36 (1.01)	5.17 (1.15)	8.087*	0.032
F4: Affect imagery	5.91 (0.75)	5.78 (0.87)	5.69 (0.85)	3.267*	0.013
F5: Mastery imagery	5.11 (0.97)	4.94 (1.07)	4.9 (0.99)	2.095	0.008
F6: Global imagery	5.52 (0.65)	5.39 (0.75)	5.37 (0.71)	6.916*	0.027

Pillai’s Trace = 0.045, *F* = 1.876, *p* < 0.05. Athletes’ gender, age and sport type are included as covariates; *Significant at the 0.05 level (two-tailed).

### 3.4 Cluster analysis: athlete achievement level group differences in imagery use

K-means cluster analysis was used to classify athletes based on their athletic achievement levels and imagery abilities scores from the SIAQ. A four-cluster solution was considered the best fit based on the number of participants in each cluster categorized by athletic achievement levels (pre-elite level, advanced level, and amateur level athlete groups) and six imagery ability scales (skill, strategy, goal, affect, mastery, and global imagery abilities). Other cluster count solutions did not show significant differences among the groups. Since Pearson correlation analysis and MANCOVA previously did not show significant differences or relationships between variables such as gender, age, and sport type, these were not included in further exploration.

To determine if the athletic achievement level groups were high or low in athletic achievements as well as imagery ability scales, a criterion z-score was adopted. The z-scores were examined and classified accordingly. Table 4 displays the mean values, standard deviation and z-scores used to create clusters. Cluster profiles are displayed in Figure 1. The classification indicated that cluster 1 had the highest overall imagery abilities, ranging from 5.85 to 6.3. Cluster 1 consisted of *n* = 185 athletes. The lowest scores in this cluster were for mastery imagery (*M* = 5.82, SD = 0.64) and strategy imagery (*M* = 6.3, SD = 0.53). The highest scores, all above 6 points, were for global imagery (*M* = 6.08, SD = 0.32), goal imagery (*M* = 6.12, SD = 0.73), skill imagery (*M* = 6.3, SD = 0.53), and affect imagery (*M* = 6.31, SD = 0.55).

**Table 4:** Mean values, standard deviation and z-scores of the four clusters.

Factor	Cluster 1 (*n* = 185)		Cluster 2 (*n* = 80)		Cluster 3 (*n* = 68)		Cluster 4 (*n* = 167)		*p*
	*M (SD)*	*z*	*M (SD)*	*z*	*M (SD)*	*z*	*M (SD)*	*z*	
F1 Skill imagery	6.3 (0.53)	0.73	5.29 (0.72)	−0.42	4.6 (0.76)	−1.23	5.57 (0.69)	−0.11	<0.0001
F2 Strategy imagery	5.85 (0.56)	0.80	4.17 (0.89)	−0.94	4.19 (0.71)	−0.92	5.01 (0.68)	−0.66	<0.0001
F3 Goal imagery	6.12 (0.73)	0.66	4.08 (0.92)	−1.14	4.28 (0.91)	−0.96	5.59 (0.75)	0.20	<0.0001
F4 Affect imagery	6.31 (0.55)	0.63	5.8 (0.51)	0.02	4.5 (0.67)	−1.56	5.73 (0.64)	−0.76	<0.0001
F5 Mastery imagery	5.82 (0.64)	0.84	4.08 (0.90)	−0.89	4.25 (0.71)	−0.72	4.77 (0.75)	−0.21	<0.0001
F6 Global imagery	6.08 (0.32)	0.99	4.69 (0.33)	−0.97	4.36 (0.36)	−1.43	5.34 (0.27)	−0.05	<0.0001

Tested by ANOVA, followed by post-hoc Tukey’s HSD, *p* < 0.05. Clusters are statistically different. K-cluster analysis algorithm successfully converged to a stable solution after 19 iterations. Z-scores were computed separately within each cluster.

Cluster 4 consisted of *n* = 80 athletes and had the second-highest imagery abilities scores. The lowest scores in cluster 4 were for mastery imagery ability (*M* = 5.34, SD = 0.27) as well as for strategy imagery ability (*M* = 5.01, SD = 0.69). Cluster 2, also consisting of *n* = 80 athletes, had imagery abilities ranging from 4.08 to 5.29. The lowest scores were for mastery imagery (*M* = 4.08, SD = 0.90), strategy imagery (*M* = 4.17, SD = 0.89), and goal imagery (*M* = 4.08, SD = 0.92). As with the previous clusters, higher scores were observed for skill imagery ability (*M* = 5.29, SD = 0.72) and with the highest scores for affect imagery ability (*M* = 5.29, SD = 0.72).

Overall, cluster 3 had the lowest scores among all clusters and consisted of *n* = 68 athletes. All scale scores were below 5 points. The lowest scores were for strategy imagery (*M* = 4.19, SD = 0.71) and mastery imagery (*M* = 4.25, SD = 0.71). The global imagery (*M* = 4.36, SD = 0.36) abilities were also lower than in other clusters, as were skill imagery (*M* = 4.6, SD = 0.76) and affect imagery (*M* = 4.5, SD = 0.67).

The ANOVA of all clusters showed significant differences between clusters regarding athletic achievement levels and imagery ability scales (*p* < 0.05). The post-hoc Tukey’s test indicated that athletes in different clusters have different levels of imagery ability.

**Figure 2:**
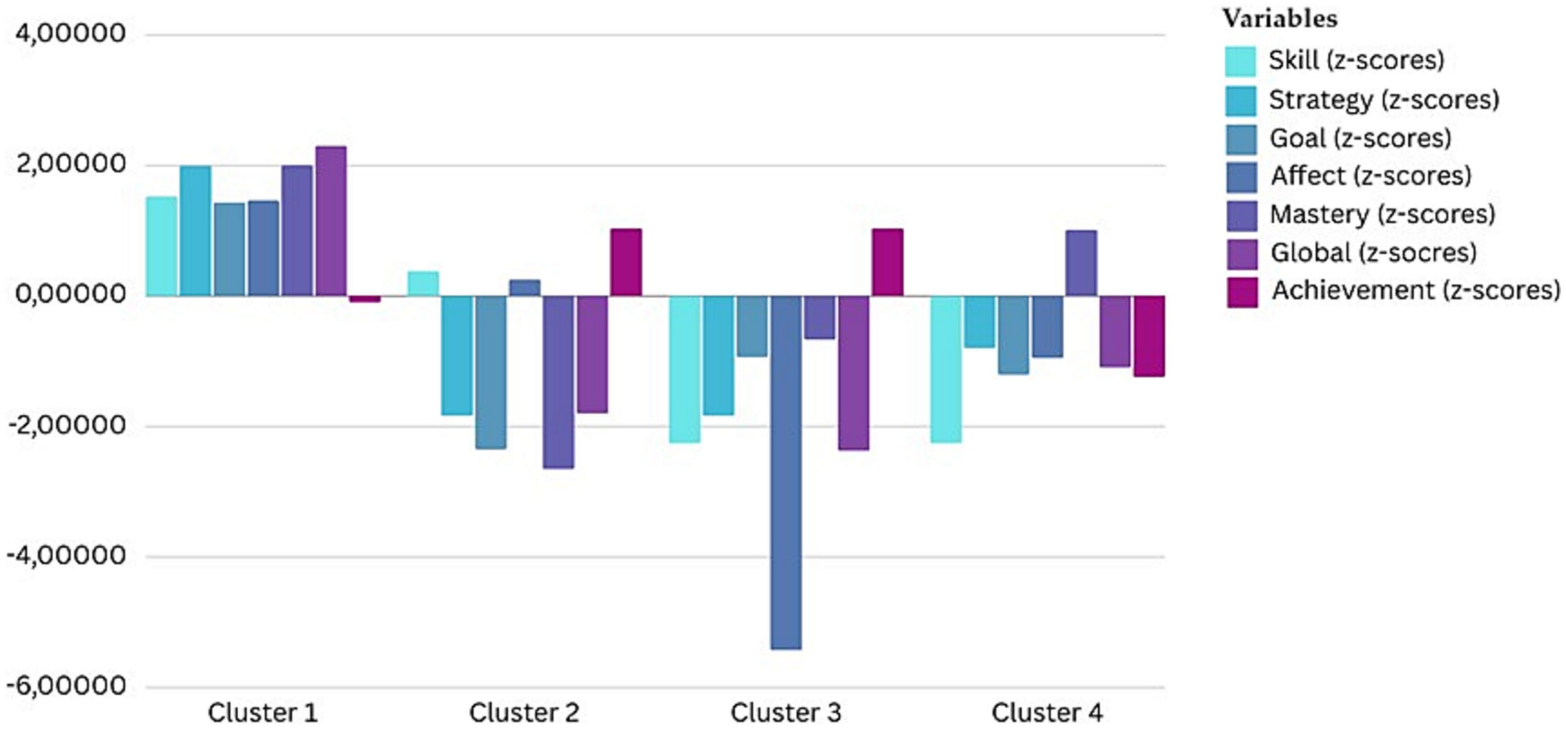
Imagery ability profiles for the four-cluster solution.

Based on the given results, the obtained clusters can be defined as follows: Cluster 1 (*n* = 185) consists of well-rounded athletes with higher achievement levels and high imagery abilities; Cluster 2 (*n* = 80) consists of athletes with average achievement levels and low imagery abilities; Cluster 3 (*n* = 68) consists of athletes with lower achievement levels and low imagery abilities; Cluster 4 (*n* = 167) consists of athletes with average achievement levels and average imagery abilities (Figure 2). The means, standard deviation as well as z-scores for all four cluster on each of the SIAQ subscales are presented in Table 4.

The significant differences shown in Table 5 between the clusters based on athletic achievement levels were confirmed (χ^2^ (4) = 42.297, *p* = 0.000). Pre-elite level athletes were less represented in cluster 2 (1%, *n* = 5), as well as advanced level athletes (3%, *n* = 15), compared to amateur level athletes (12%, *n* = 60). Similarly, pre-elite level athletes were less represented in cluster 3 (3%, *n* = 15) and advanced level athletes (3.8%, *n* = 19) compared to amateur level athletes (6.8%, *n* = 34). Cluster 4 represents the most pre-elite level athletes (18.6%, *n* = 93), along with a significant number of advanced level athletes (7.8%, *n* = 39) and amateur level athletes (7%, *n* = 35). Cluster 1 consists of the second largest number of pre-elite level athletes (11.6%, *n* = 58), as well as the largest number of advanced level athletes (8.4%, *n* = 43) and amateur level athletes (17%, *n* = 185).

**Table 5:** Athletes cluster distribution according to athletic achievements levels.

	Cluster 1		Cluster 2		Cluster 3		Cluster 4		*p*
	*n*	*%*	*n*	*%*	*n*	*%*	*n*	*%*	
*Achievement level*									<0.0001
*Pre-elite level*	58	11.6	5	1	15	3	93	18.6	
*Advanced level*	42	8.4	15	3	19	3.8	39	7.8	
*Amateur level*	85	17	60	12	34	6.8	35	7	
*Total*	185	37	80	16	68	13.6	167	33.4	

Statistical significance assessed using Pearson Chi-Square = 42.297, df = 4, *p* < 0.01 (two-sided).

### 3.5 Experiment: impact of guided imagery intervention on athletes’ performance outcomes

The data obtained in the experimental part of the study were analyzed for nine alpine skiers, selected based on previously defined criteria for pre-elite and advanced-level athletes. The experiment aimed to empirically test the relationship between the cluster imagery profile and athletic achievements.

Table 6 presents the scores for the scales of imagery abilities of the sample before and after the guided imagery intervention. The data reveal a similar trend to that observed in the cluster analysis, where the highest scores for imagery abilities were in affect imagery (*M* = 5.27; SD = 0.79), skill imagery (*M* = 4.84; SD = 0.77), and mastery imagery (*M* = 4.90; SD = 1.04) ability. The lowest scores were found in strategy imagery ability (*M* = 3.94; SD = 0.88) and followed by goal imagery (*M* = 4.40; SD = 1.61) ability. The indicators for this sample closely match the second cluster, which consists of mid-level athletes with lower imagery abilities. Given that the sample comprises youth athletes, their abilities also align with the average or advanced levels. The global imagery abilities (*M* = 4.66; SD = 0.71) correspond to the second cluster.

**Table 6:** Assessments of athletes’ imagery ability scores before and after guided imagery intervention (*n* = 9).

Imagery ability	Before		After		Wilcoxon signed ranks test	
	*M*	SD	*M*	SD	*Z*	*p*
Skill imagery	4.84	0.77	5.27	0.73	−1.838	0.066
Strategy imagery	3.94	0.88	4.63	0.83	−2.028	0.043*
Goal imagery	4.4	1.61	4.81	1.37	−1.781	0.075
Affect imagery	5.27	0.79	5.64	0.52	−2.254	0.024*
Mastery imagery	4.9	1.04	4.9	0.93	−0.106	0.916
Global imagery	4.66	0.71	5.03	0.67	−2.668	0.008*

*Significant at the 0.05 level (two-tailed).

Following the inclusion of the guided imagery intervention in the training process, there was a positive increase in most scales of imagery abilities, with the exception of mastery imagery, where the increase was not noticeable. A Wilcoxon Signed Ranks Test was employed to evaluate differences in imagery abilities scores of the SIAQ imagery ability scales before and after the guided imagery intervention. The results indicated statistically significant differences in strategy imagery ability (*Z* = −2.028, *p* = 0.043, *r* = 0.68; where r – effect size for the Wilcoxon signed-rank test; *p* < 0.05), affect imagery ability (*Z* = −2.254, *p* = 0.024, *r* = 0.75), and global imagery ability (*Z* = −2.668, *p* = 0.008, *r* = 0.89). Other scales showed an increasing trend, but the changes were not statistically significant.

**Figure 3:**
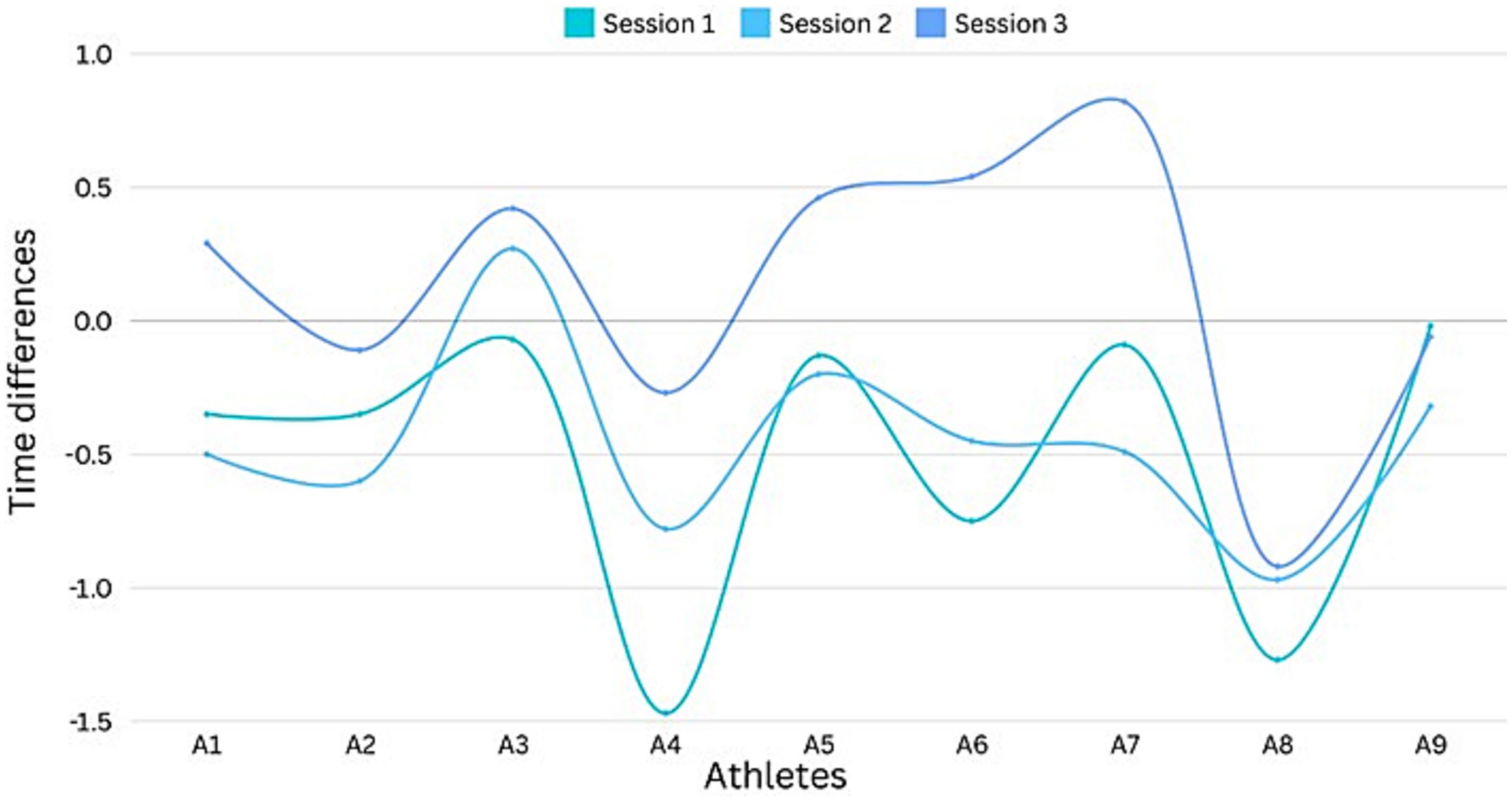
The outcomes of the control training sessions. Negative values indicate a faster second run.

Figure 3 illustrates the dynamics of control training session results for alpine skiers. The graph illustrates the time differences for alpine skiers between their first and second successful attempts on the same slalom course. Both runs were conducted on the same ski setting. A negative time difference indicates that the second run was faster than the first, suggesting that the athlete may have skied more cautiously during the initial attempt, possibly due to psychological factors such as fear of making mistakes. Conversely, a positive time difference signifies that the first run was faster than the second.

The results of the first control training show that the time differences ranged from −1.27 s to −0.02 s (*M* = −0.5; SD = 0.54). In the second control training, the range narrowed to between −0.97 and −0.2 s (*M* = −0.45; SD = 0.35). By the third and final control training, the time differences ranged from −0.92 to 0.82 s (*M* = 0.13; SD = 0.53).

The reliability of the changes in the average time differences was assessed using the Wilcoxon signed-rank test. No significant changes were observed between the first and second control training sessions (*Z* = −0.416, *p* = 0.677, *r* = −0.139). However, statistically significant improvements were noted between the second and third control training sessions (*Z* = −2,666, *p* = 0.008, *r* = −0.889), as well as between the first and third sessions (*Z* = −2,547, *p* = 0.011, *r* = −0.849).

At the beginning of the study, prior to the integration of guided imagery session, the obtained results indicate that athletes had difficulties to show their best performance from the very first run. The faster times in the second run indicate that the athletes had the potential to complete the course more quickly but were unable to fully capitalize on this potential. This underscores the importance of mental training and highlights the inadequacies in psychological preparation. It can be concluded that the intervention of guided imagery have a positive effect on the performance of athletes. Incorporating imagery into the athletes’ process contributes to their overall performance improvement and supports their positive development.

### 3.6 Practical recommendations for enhancing imagery ability in the promotion of achievements for athletes based on K-means cluster analysis and experiment results

Based on the results of the k-means cluster analysis, the characteristics of the SIAQ scales, findings from the experimental part of this research, and practical experience, it is possible to propose recommendations for the development of each type of imagery ability and its associated functions. Each imagery ability exhibits slightly differentiated content, and this differentiation should be a key focus for athletes during training as they work to develop specific imagery skills. Strategy imagery, in particular, serves as a crucial foundation for the growth and development of athletes. It encompasses an athlete’s ability to manage and employ guided imagery to regulate their pre-competition state and overall preparation for competition. To enhance these abilities, the daily training process should incorporate competitive elements and guided imagery techniques aimed at regulating emotional states and activating internal resources. These practices assist athletes in mentally preparing before competitions and analyzing their performance afterward. The indicators of these imagery ability offer insights into how successfully an athlete can generate alternative strategies in their imagery to address unexpected and challenging situations that may arise under high-pressure conditions. Strategy imagery plays a vital cognitive role, facilitating the learning, refinement, and maintenance of strategies, competitive plans, and routines.

Mastery imagery ability reflect the level of integrative capabilities an athlete possesses. This ability is characterized by the athlete’s capacity to visualize themselves performing at 100% effort, even when things go wrong, or mistakes are made. Athletes with strong mastery imagery ability can maintain their fighting spirit after setbacks and stay focused on their subsequent performance, while also preserving self-efficacy and confidence in challenging situations. During training, a key focus should be placed on maintaining full effort, especially in the final 15 min of intense training, even when errors occur. Afterward, athletes should learn to visualize and cultivate a “feeling of success and power” within themselves. This practice helps them maintain emotional control, self-confidence, fighting spirit, and the ability to transform negative “inner speech” into positive affirmations in various unexpected competition situations. Mastery imagery serves a motivating general-mastery function, enabling athletes to make desired modifications within their imagery. It allows them to manage and manipulate the images and content created in their minds. It should be noted that this level of imagery reflects a highly developed skill that has been consistently integrated into the athlete’s daily training process over a long period. However, the study results indicate that mastery imagery ability is one of the least expressed and most difficult abilities to develop among the athlete population.

Goal, affect, and skill imagery ability form the foundation for developing strategic and mastery imagery ability. Goal imagery specifically refers to the athlete’s ability to create mental images associated with achieving specific goals. This ability allows athletes to feel like winners without losing focus on the process. Goal imagery ability is closely linked to an confidence and self-efficacy. Understanding and characterizing these abilities in sports can help evaluate and draw conclusions about an athlete’s self-efficacy, motivation, and satisfaction with their performance. Goal imagery ability serves the motivational-specific function of imagery. To develop and effectively apply these abilities in competition, athletes must learn to set clear, time-bound goals and outline the steps needed to achieve them during daily training. For example, an athlete might set a goal to improve the result of a particular technical performance within 2 weeks. These goals can pertain to various aspects—technical skills, physical attributes, emotional regulation, or other areas. Over time, the consistent practice of goal setting, supported by this specific function of imagery, will become habitual. This function plays a crucial role in guiding specific sports processes, enhancing performance, and achieving goals.

Skills imagery ability enables athletes to use their imagery during training to facilitate the learning of new technical tasks or elements. Once this form of imagery ability is automated, it helps athletes internalize the sensations of their body when performing specific technical elements. It also aids in error correction and technique improvement. To some extent, skills imagery also enhances the effective use of imagery in competitive environments. It is particularly valuable in training for learning and executing new technical exercises, developing physical abilities, and refining technical skills. Often, it serves as a powerful teaching tool in the training process. Skills imagery ability fulfills a cognitive-specific function, ensuring the acquisition, improvement, and maintenance of specific skills.

On the other hand, affect imagery ability equip athletes with the essential skills for emotional self-regulation and are fundamental to maintaining physical self-efficacy. These abilities help athletes understand and manage the emotions experienced during both training and competition. They allow athletes to visualize the feelings and emotions that arise from their performance results, aiding in the regulation of anxiety. Additionally, affect imagery enables athletes to anticipate the emotions associated with achieving specific competitive outcomes and to experience the excitement of competition. This, in turn, helps them mentally prepare and focus for future competitions. Affect imagery ability serves the motivational general-affective function of imagery, playing a key role in regulating emotions, mood, arousal, and anxiety. In sports, this imagery ability is strongly correlated with physical self-efficacy, as supported by research in sports science.

## 4 Discussion

One of the primary objectives of this study was to evaluate the interrelationships between individual differences in imagery abilities among athletes of different achievement levels. More specifically, the study aimed to determine whether the different imagery profiles identified through cluster analysis reflect the achievements of athletes. Cluster analysis was employed in a non-biased manner, avoiding arbitrary labeling of results as excessively high or low. To maximize the accuracy and relevance of the imagery ability profiles in the sample group, k-means cluster analysis was selected as the most appropriate method. K-means cluster analysis offers a valuable opportunity to categorize athletes, in this case based on their athletic achievements and imagery abilities. This approach allows for the categorization of athletes based on their athletic achievements and imagery abilities, providing a deeper understanding of how these factors are distributed and how they relate to performance.

The question of how imagery, as a psychological skill, can influence athletes’ achievements has been a central topic in sports research for many years. However, empirical studies conducted over recent years have yet to reach a consensus, especially when considering athletes across different performance levels. For instance, Dello Iacono et al. (2021) concluded in their research on mental imagery in high-performance sports that mental imagery training has proven to be an effective tool for maintaining and improving athletic performance, particularly among pre-elite level athletes. Similarly, Hartani and Yang (2023) found in their study on the impact of imagery on skill acquisition and sports performance that imagery has a significant and reliable effect on sports achievement, highlighting its critical role in athletic success.

In the present study, the research sample of 500 athletes was grouped into four clusters based on their athletic achievements and imagery abilities. Creating such psychological profiles among athletes allows for the scientific categorization of athletes into groups based on these factors, thereby enhancing our understanding of how psychological aspects are related to athletic achievements. Cluster 1 (high achievement and high imagery abilities) suggests that athletes in this group could benefit from targeted interventions that focus on maintaining and further developing their psychological skills, particularly in mental imagery, to sustain their high level of achievement. Cluster 2 (average achievement and low imagery abilities) indicates that these athletes could see significant improvements if substantial effort is invested in enhancing their psychological preparation, particularly their imagery abilities, which in turn could boost their physical performance. Cluster 3 (low achievement and low imagery abilities) highlights the need for a dual focus on both physical skill development and psychological readiness. Enhancing imagery abilities could be crucial in overcoming the performance limitations faced by this group. Cluster 4 (average achievement and average imagery abilities) suggests that while these athletes are relatively balanced, they could benefit from they could benefit from a holistic approach to their training. Focusing on physical and mental development may enhance their performance levels. The identification of these clusters offers a clear starting point for developing tailored interventions based on athletes’ unique athletes profiles.

Based on the results of the k-means cluster analysis, which provided significant insights into the profiles of athletes’ imagery abilities at various levels of athletic level achievement, the study was able to develop an individually tailored guided imagery intervention for integration into the training process of selected alpine skiers. This intervention aimed to enhance the athletes’ imagery abilities. The secondary aim of the study followed from this: to evaluate the effectiveness of the guided imagery intervention on the imagery abilities and athletic performance of a sample of nine alpine skiers during control training sessions, thereby offering insight into potential performance improvements. The influence of imagery on athletic performance and its enhancement has been extensively explored in the literature (Lee et al., 2023; Hartani and Yang, 2023; Lochbaum et al., 2022). However, fewer studies have taken a holistic approach to developing athletes’ overall psychological skills.

In sport psychology, imagery abilities are often assessed based on two key criteria which are the ease or difficulty of imagining a specific situation, and the vividness of the imagery (Cumming et al., 2023; Bedir and Erhan, 2021). These criteria are essential for determining an athlete’s effectiveness in utilizing imagery techniques. Given the complex nature of imagery, experimental research plays a crucial role in bridging the gap between theoretical concepts and practical applications in sports techniques (Cumming and Quintom, 2022). Imagery is widely recognized as an effective technique for enhancing learning, performance, and rehabilitation after injury. However, evidence on its overall effectiveness has been mixed (Simonsmeier et al., 2021). This inconsistency highlights the importance of focusing on key factors for successful imagery, such as creating and controlling vivid, realistic mental images.

The guided imagery intervention employed in this study demonstrated a positive effect on both the athletes’ imagery abilities and their performance in control training sessions. This suggests that individualized imagery ability development approach is a key factor in enhancing athletes’ psychological and physical performance. The results suggest that guided imagery interventions not only enhance imagery abilities but also positively impact athletes’ performance, with statistically significant improvements observed. However, no significant change was found in mastery imagery, suggesting that this specific aspect of imagery ability may require a different approach or a longer intervention period to show measurable improvements. Such findings align with previous research, emphasizing the value of personalized psychological interventions in athletic development (Hartani and Yang, 2023).

The ability to use imagery effectively can be developed through visualizing various events and situations and by engaging with different emotions, as suggested by several researchers (e.g., Lu et al., 2020; Predoiu et al., 2020). Other researchers argue that imagery allows athletes to mentally prepare for competitions, thereby optimizing their psychological readiness (Janjigian, 2024). For athletes to execute desired actions mentally, they must cultivate imagery skills that enable them to create and maintain vivid and controllable images for a sufficiently long period. Developing these abilities is a highly individualized process that requires a tailored approach for each athlete. Individual differences in imagery ability significantly affect an athlete’s learning, performance, and cognitive outcomes in sport. These differences can be influenced by factors such as image controllability, and previous experiences, which researchers and sports professionals cannot fully control. Consequently, these factors greatly influence the content and effectiveness of an athlete’s imagery. To derive the full benefit from psychological skills development, athletes must learn to effectively utilize their imagery and harness its potential. This personalization of mental skills is one of the key factors driving successful sports interventions, as emphasized in various studies.

The PETTLEP model (Holmes and Collins, 2001) has become a widely accepted framework for enhancing imagery effectiveness. Developed over 20 years ago, has become a standard reference point for many practical and theory-based imagery techniques in sports. This model emphasizes components to enhance the effectiveness of imagery practices. Recent empirical studies suggest that imagery training should be as individualized as possible, adapting to the unique characteristics of each athlete to maximize effectiveness (Simonsmeier et al., 2021). As such, future research should continue to explore individualized imagery strategies and consider how different factors impact an athlete’s ability to visualize and mentally rehearse sports scenarios.

Based on the results of this study and the existing literature, the practical applications are clear. The findings provide a basis for identifying and implementing targeted interventions in psychological skills and imagery for athletes. Such kind interventions can help athletes unlock their full potential and enhance their athletic performance. Many research has empathized that imagery plays a significant role in improving various aspects of sports performance, such as skill execution, motivation, and self-confidence (Cumming et al., 2023; Lee et al., 2023). From a practical perspective, sports coaches and psychologists can use the results of this study to allocate resources more effectively. For example, athletes in clusters with lower imagery abilities might require more psychological support, while those in higher-achieving clusters might focus on maintaining their competitive edge (Lochbaum et al., 2022).

Additionally, this study can inform talent identification and development programs by highlighting key attributes that contribute to athletic success. This is especially relevant given that the study includes pre-elite level athletes, providing insights into trends within the sports environment in Latvia. Research indicates that early identification of psychological attributes, such as imagery ability, can enhance long-term performance outcomes (Simonsmeier et al., 2021). By identifying groups of athletes with similar characteristics it allows for the development of individualized psychological interventions that meet the specific needs of each athlete. This contributes to the advancement of sports science and the creation of more effective training methods.

The SIAQ scales provide a framework for understanding athletes’ imagery abilities. However, the scientific literature lacks practical and specifically oriented instructions on how to promote growth in each scale. Addressing this gap was a crucial step in the development of this research, achieved through k-means cluster analysis. Using the significance of the SIAQ scales, cluster analysis results, and a comprehensive literature review, this study developed a guided imagery intervention. The purpose was to test this intervention’s effectiveness in controlled training environments to enhance alpine skiers’ athletic performance. A sample of alpine skiers, comprising pre-elite and advanced-level athletes, participated in 47 guided imagery session exercises systematically integrated into their training routines. This approach aligns with the arguments of researchers such as Blumenstein and Orbach (2020), who emphasize that psychological preparation should be integrated with physical training and competition processes. The results of the implemented intervention suggest that this kind of approach can have a positive impact on athletes’ achievements and physical performance, highlighting the essential role of imagery in both psychological preparation and physical execution.

Previous research also shows positive and significant relationship between athletes’ use of imagery and their performance. For example, Lin et al. (2021) argued that emphasizing strategic and technical imagery training for athletes could enhance performance, support goal achievement, and improve overall satisfaction with the sports process. These findings suggest that incorporating imagery benefits not only psychological preparation but also physical training. A review by Ladda et al. (2021) affirmed that the practice of imagery in sports leads to effective outcomes in both psychological and physical preparation. Similarly, other research has found that imagery training increases athletic performance and reduces negative psycho-emotional states, enabling athletes to cope more effectively with stress, which significantly impacts physical performance (Williams et al., 2021; Yadolahzadeh, 2020).

Overall, research in sports science has highlighted the importance of psychological factors, such as imagery, in athletes’ preparation and achieving high performance. A deeper understanding of imagery as a psychological factor and the imagery profiles of different sports groups can significantly aid in developing training programs. Furthermore, incorporating psychological interventions in sports can enhance athletes’ mental resilience, a crucial aspect of modern sports. The practical application of this study’s findings suggests that integrating structured imagery training into athletes’ regular training practice routines can significantly enhance their overall performance. By evaluating athletes’ imagery abilities, coaches and sports psychologists can design targeted psychological skills interventions tailored to individual needs based on their imagery profiles and level of achievement. For instance, high-achieving athletes with strong imagery abilities may benefit from advanced visualization techniques, while lower-achieving athletes may require foundational imagery training to develop their skills. Additionally, the study underscores the critical role of affective imagery in psychological training, particularly in the demanding modern sports environment. The demonstrated effectiveness of guided imagery interventions among alpine ski athletes further highlights the value of incorporating these techniques into training programs to optimize mental preparation and athletic success.

### 4.1 Limitation of the study

While this study employed a validated and widely recognized imagery ability measurement tool in sports psychology research, several important limitations should be acknowledged when interpreting the results. One key limitation is that imagery ability was assessed using a self-reported measurement tool, which may introduce common method variance. However, given the complexity of psychological variables like imagery ability, self-reported data might be the most valid measurement available for this study. Additionally, the Sport Imagery Ability Questionnaire (SIAQ) has demonstrated its validity and reliability across various contexts within sports psychology and imagery ability research over the past decade. The large sample size of 500 athletes, each with varying and notable achievements, is also a strength within the framework of this research.

Another limitation to consider is the two-year time frame during which the data were collected. Data were gathered at different phases of the training seasons, which could have influenced athletes’ responses and the evaluation of their imagery abilities. For instance, during the off-season, athletes might focus more on imagery skills related to the training process rather than those linked to competition or mastery. These varying circumstances could have affected the study’s findings.

It is also important to acknowledge the limitations of the experimental part of the study. The main limitation is that the guided imagery sessions were conducted with a small sample of alpine ski athletes, so it is not possible to generalize the data to the whole athlete population. Additionally, the study lacked a control group, which limits the ability to determine causality and fully isolate the effects of the guided imagery intervention. Future research should aim to expand the sample size and include athletes from a broader range of sports. This would offer a more comprehensive understanding of the psychological preparation process across different athletic disciplines. This study is significant because it enhances our understanding of the role of imagery abilities in athletes’ achievements. By confirming the critical role of imagery, this study highlights the potential benefits of prioritizing and cultivating imagery abilities in athletes. Future research should analyze imagery abilities in conjunction with other psychological variables. This would provide a broader understanding of the different aspects of psychological skills and could lead to more objective identification of clusters and outcomes.

## 5 Conclusion

The relationship between differences in imagery ability and athletic achievements across varying levels of athletes has been understudied. This study demonstrates a clear link between these factors. The findings suggest that imagery ability is significantly related to athletic performance, highlighting the potential value of incorporating imagery training into athletes’ regular training process and routines. Measuring imagery ability in athletes could be highly beneficial for practical research, as it would allow not only for the observation of how frequently athletes use imagery but also for understanding the specific purposes for which they employ it across different skill levels. This information could be instrumental in planning and selecting appropriate content for psychological skills interventions tailored to athletes’ needs.

The study’s results reveal a significant and statistically reliable correlation between skills, strategy, goals, affect, and global imagery abilities among the athletes sampled. The only exception was mastery imagery, although mastery imagery was still reliably correlated with other imagery abilities, suggesting that higher-achieving athletes possess higher level of imagery abilities overall. No significant differences in imagery abilities were observed between team and individual sports. Similarly, the long-debated issue of gender differences in sports revealed no significant differences in mean scores, except for one finding: women displayed significantly higher affect imagery abilities, particularly those focused on emotions.

The analysis led to the creation of four clusters based on athletes’ imagery ability profiles and their achievements in sports. These clusters indicate that typical imagery ability profiles differ according to athletes’ levels of achievement. Cluster 1, comprising high-achievement athletes, demonstrates strong imagery abilities. Cluster 2 consists of athletes with average success in sports and lower imagery abilities. Cluster 3 confirms that low-achieving athletes have weaker imagery abilities, while Cluster 4 represents athletes with average achievements and average imagery abilities. These insights enable the design of effective psychological skills interventions tailored to the specific needs and abilities of athletes based on their imagery profiles.

Including a sample of nine alpine skiing athletes from Cluster 2 in the experiment reinforced previously established insights on the value of imagery as a psychological skill in sports, particularly in the psychological preparation of athletes and overall performance. The results indicated that the guided imagery intervention significantly enhanced the imagery abilities of the athletes across all SIAQ scales, apart from mastery imagery ability. Control training sessions also resulted in positive and statistically significant improvements in athlete performance, highlighting a clear interrelationship between imagery and athletic performance.

## Data Availability

The datasets presented in this study can be found in online repositories. This data can be accessed at DOI: 10.48510/FK2/W2Z2PY (Volgemute, 2025).
