# Supplementary material for: The benefits of guided imagery on athletic performance: a mixed-methods approach
Source: Front Psychol. 2025 Apr 11;16:1500194. doi: 10.3389/fpsyg.2025.1500194 (PMC12021890; doi:10.3389/fpsyg.2025.1500194)
Supplement: Supplementary file 1 [file Table_1.docx]

Supplementary Material

# Supplementary Figures

*Example of guided imagery training session plan on-snow*

| **Action nr.** | **Aim** | **Guided imagery training session** | **Time**  **(min)** | **Outcome** |
| --- | --- | --- | --- | --- |
| ***1.*** | ***Introduction*:** Developing awareness of the practical application of imagery on-snow training. | Remind athletes to the concept of guided imagery. Emphasize the role of imagery and highlight the essential keys to its effectiveness: engaging the senses (sight, sound, touch, smell), using different perspectives (internal/external), creating a realistic experience, and maintaining self-control throughout the process. | **2** | Athletes understand the concept of imagery and realize its value in alpine ski competitive sport. |
| ***2.*** | ***The main part:*** Learn and continue to integrate guided imagery into on-snow training. | Guided imagery training is integrated into the on-snow training process of alpine skiers. | **15** | Athletes' imagery ability is developed through the application of practical exercises of imagery on-snow training.  **Time recording protocol:**  *Mental Time:*  *Run 1 ______ sec*  *Run 2 ______ sec*  *Real time:*  *Run 1 ______ sec*  *Run 2 ______ sec*  *In this exercise, it is ideal for the athletes to match their mental run times as closely as possible in each attempt.* |
| *2.1.* |  | A slalom course is prepared on the snow for the athletes. After performing their physical warm-up exercises, the athletes walk through the course for inspection. At this point, the course is not skied physically; rather, athletes focus on observing the course layout, noting key turns, and mentally preparing for the run. |  |  |
| *2.2.* |  | Following the inspection, athletes engage in a guided imagery exercise. Here, they visualize completing the slalom run, imagining each turn, terrain change, and movement. This "introductory" run is done entirely in their minds. The coach times this mental run to track how long it takes the athlete to mentally complete the course. |  |  |
| *2.3.* |  | Once the athletes complete their mental run, they perform the actual physical skiing run on the course. The time taken to complete the real run is recorded. The coach then compares the mental and physical times, with the objective of having the mental time closely match the physical time. This process of mental run followed by a physical run is repeated for consistency and practice. |  |  |
| ***3.*** | ***The Concluding part:*** Training session summary | Athletes reflect on the task performed. | **3** | Feedback is provided that strengthens awareness of the conditions for applying imagery in training and competition. |
